# The Development and Testing of the Hippocratic Heart Failure Self-Care Scale

**DOI:** 10.3390/healthcare12080820

**Published:** 2024-04-11

**Authors:** Hero Brokalaki, Anastasia A. Chatziefstratiou, Nikolaos V. Fotos, Athina Patelarou, Konstantinos Giakoumidakis

**Affiliations:** 1 Department of Nursing, School of Health Sciences, National and Kapodistrian University of Athens, 11527 Athens, Greece; heropan@nurs.uoa.gr (H.B.); nikfotos@nurs.uoa.gr (N.V.F.); 2 Cardiac Surgery Unit, General Peadiatric Hospital of Athens “Agia Sophia”, 11527 Athens, Greece; a.chatziefstratiou@yahoo.gr; 3 Department of Nursing, School of Health Sciences, Hellenic Mediterranean University, 71410 Heraklion, Greece; apatelarou@hmu.gr

**Keywords:** self-care, self-management, heart failure, validation, scale development

## Abstract

Background: The adoption of self-care behaviors among patients with congestive heart failure (CHF) is essential for the management of their health condition. However, there is a lack of tools for estimating self-care in CHF patients. We aim to develop and validate the Greek version of the Hippocratic heart failure self-care scale (HHFSCS). Methods: The scale includes 22 items which are reviewed by a committee of experts. Individuals indicate the frequency at which they follow each self-behavior on a five-point Likert scale. Adult patients with CHF (*n* = 250) from a General Hospital in Athens participated in the study from June 2020 to March 2021. Reliability coefficients and an explanatory factor analysis (EFA), using a Varimax rotation and the principal component method, were used to assess the psychometric measurements. Results: The Cronbach’s alpha coefficient of the HHFSCS was 0.807. The exploratory factor analysis identified two domains that accounted for 88.44% of the variance in the scale items; however, each sub-scale could not be used as an independent scale. Finally, the test–retest showed a significant and strong correlation (r = 0.973, *p* < 0.001). Conclusions: The HHFSCS is a reliable and valid tool for assessing self-behaviors in CHF patients. Health professionals can use it in their clinical practice to improve the management of a patient’s health conditions.

## 1. Introduction

Chronic heart failure (CHF) is a pervasive and complex chronic syndrome impacting individuals worldwide. As reported by the American Heart Association, the prevalence of CHF among Americans aged 20 and older was approximately 6 million between 2013 and 2016, whereas between 2009 and 2012, it was almost 5.7 million. Notably, there was a significant increase of 5.7 million cases between 2009 and 2012, with a projected further increase of 46% from 2012 to 2030, reaching over 8 million individuals over 18 years old. This increase indicates that the incidence of CHF is expected to rise from 2.42% in 2012 to 2.97% in 2030 [1]. The European Society of Cardiology also notes that in developed countries, the incidence of CHF is estimated at 1–2% among adults but rises to nearly 10% among those aged over 70. Additionally, the risk of CHF is 33% for men at the age of 55 and 28% for women [2]. In Greece, it is believed that about 200,000 patients have CHF, with around 30,000 new diagnoses reported every year [3].

Individuals with CHF often experience poor clinical outcomes, resulting in frequent hospitalizations. In Greece, the hospitalization rate is estimated at 19%, with an annual mortality rate of 8% during one year of follow-up. However, patients with a history of previous CHF-related hospitalizations exhibit higher hospitalization and mortality rates, at 42% and 24%, respectively [4]. The high rate of hospitalization is related to an important rise in the total healthcare cost. Regarding Greece, Parisis et al. [5] found that hospitalization for CHF accounts for 75% of the total cost related to CHF, amounting to approximately EUR 2300 to EUR 3200 per hospitalization. In 2012, the estimated worldwide cost of CHF was USD 533 million (approximately €416 million) [6]. However, the direct cost of CHF exceeds EUR 4400 per person annually in Greece [4].

It is essential to highlight that a significant portion of hospitalizations and costs associated with CHF are preventable and can be avoided, primarily attributed to low adherence to the recommended therapeutic regimen. The low level of medication adherence is associated with increased symptom manifestation. Also, patients often visit a hospital because they are not able to see any changes in the signs and symptoms of their chronic disease. These imply that patients often choose not to follow the healthcare professionals’ prescribed instructions and fail to adopt the suggested self-care behaviors necessary for effective condition management [4,5].

In 2003, the European Society of Cardiology defined self-care as “the decision and strategies undertaken by the individual to maintain life, healthy functioning, and well-being”. Self-care behavior can be health-deviated or developmental, depending on whether it is needed by every person, emerges from health issues, or is associated with a particular period in life [7]. In 2021, the European Society of Cardiology emphasized the significance of effective patient self-care in managing heart failure, leading to better quality of life, decreased readmission rates, and lower mortality rates [2,8].

However, the literature review reveals a lack of instruments and tools for assessing self-care behaviors among CHF patients, which are crucial for healthcare professionals to develop and implement strategies for improvement. For instance, the Self-Care Assessment Schedule (SCAS) was developed by Burnes and Benjamin and assesses ten self-care behaviors during a 14-day period of time; however, it is not a disease-specific tool for CHF [9]. The Self-Care Behavior Questionnaire was developed by Dodd in 1984 to estimate self-care among patients with cancer who face side effects of chemotherapy [10]. The Self-Care in Chronic Illness Questionnaire includes 45 items, and it is not a disease-specific questionnaire for CHF [11].

On the other hand, we found some tools assessing self-care among patients with CHF; however, they are characterized by some limitations. First of all, the Beliefs about Medication and Compliance Scale and the Beliefs about Dietary Compliance Scale were developed by Bennett et al. [12]. Both of these two scales aim to assess patients’ beliefs about the benefits of and barriers to medication and diet adherence in patients with CHF. The Self-Care of Heart Failure Index is a disease-specific instrument that evaluates self-care behaviors in CHF [13]. It includes 15 items sub-divided into three scales. More specifically, the Self-Care of Heart Failure Index assesses self-care maintenance, self-care management, and self-care self-confidence. Self-care maintenance concerns symptom monitoring and treatment adherence so that patients are able to adopt a healthy lifestyle. Self-care management is a dynamic, intentional decision-making approach initiated in response to symptoms. Self-care management is based on symptom recognition, symptom evaluation, and treatment evaluation, which are related to self-efficacy. In other words, patients should be able to recognize any change in the signs and symptoms of CHF and respond immediately. Finally, the scale assesses self-care maintenance based on CHF clinical guidelines regarding diet, body weight, exercise, and flu vaccination, whereas the questions related to self-care management are about signs and symptoms of CHF.

The last disease-specific tool for CHF is the Revised European Heart Failure Self-Care Behavior Scale, which was published in 2003 and has been translated into many languages [14]. The scale includes 12 items and assesses self-care behavior in patients with heart failure over time. More specifically, the items negotiate patients’ self-care regarding body weight, symptom management, flu vaccination, exercise, diet, and medication adherence. However, according to the analysis, three items were excluded from the scale which are very significant issues in patients with heart failure. These items refer to “taking rest if dyspnea occurs”, “flu shot”, and “medication adherence”.

The recognition of symptoms and signs of heart failure and the knowledge of their management are essential issues in the management of heart failure. Healthcare providers educated patients and their families about the symptoms of heart failure, like dyspnea, fatigue, and edema, and how to manage them. Therefore, it is an important scale for assessing how patients face the symptoms of their health condition since the ineffective management of their symptoms leads to a deterioration in their quality of life. Also, healthcare workers should be able to identify any possible gaps in the knowledge of their patients to provide them with appropriate education. Moreover, flu vaccination is an important part of the management of CHF since patients with heart failure are at high risk when they contract influenza. However, the researchers excluded this item because of its psychometric properties. The last deleted item is related to medication adherence. Medication adherence plays a significant role in the management of all chronic diseases like heart failure, and it is an integral part of self-care. From all of the above, it is obvious that the Revised European Heart Failure Self-Care Behavior Scale does not include important aspects of the self-care behavior of patients with heart failure.

Therefore, the primary objective of this study is to develop and test the Hippocratic heart failure self-care scale. Specifically, the prevailing study goals are as follows:

- To develop the Hippocratic heart failure self-care scale.
- To evaluate the psychometric proprieties of the Hippocratic heart failure self-care scale through reliability and exploratory factor analyses (EFAs).

## 2. Materials and Methods

### 2.1. Study Design

The development of the Hippocratic heart failure self-care scale involved a comprehensive literature review of recent data and reports from health associations such as the European Society of Cardiology [2,8]. A 30-item scale was created, comprising 8 sub-sections: medication aspects (items 1–6), diet aspects (items 7–15), exercise aspects (items 16–17), alcohol aspects (items 18–19), smoking aspects (items 20–22), symptoms (items 23–26), appointment keeping (items 27–28), and vaccination aspects (items 29–30). Each item was presented as a full sentence and rated on a five-point Likert scale from “never” (0 points) to “very frequently” (4 points), resulting in an entire score range of 0 to 120.

To assess content validity, the opinions of seven experts, including cardiologists, heart failure-specialized nurses, statistics experts, and psychometrics experts, were solicited through an evaluation form. The task force categorized each item as “essential”, “useful but inadequate”, or “unnecessary”. Their feedback was incorporated into the scale, leading to the exclusion of 8 items due to overlap between sub-sections. The clarity of all items was also evaluated and refined with input from 50 non-CHF individuals without research backgrounds.

Ultimately, the Hippocratic heart failure self-care scale was reduced to a 22-item scale with 8 sub-sections. Table 1 presents the total scale. Items 1–4, 6–8, 11, 13–16, and 19–22 were reverse-scored. Points over 52 were classified as “very good”, 48–51 as “good”, 43–47 as “fair”, and below 42 as “poor” based on score quartiles. Therefore, a higher score indicates better self-care behavior among patients with heart failure.

### 2.2. Study Population

The present research was carried out at a General Hospital in Athens from June 2020 to March 2021. In total, 250 men and women were hospitalized in the Cardiology Unit due to either deteriorating health conditions or scheduled procedures. The sample size was calculated so that the question item/participant ratio would be at least 1/10 [15]. The inclusion criteria included being at least 18 years old, exhibiting symptoms of CHF NYHA II-IV, having a confirmed CHF diagnosis based on ultrasound (HFrEF), having the ability to read and write Greek, having written informed consent received, the absence of life-threatening diseases other than CHF, the absence of psychiatric disorders, no cardiac surgery within the last 6 months, and no musculoskeletal disorders affecting physical activity.

### 2.3. Assessments

Data collection involved face-to-face interviews during the initial assessment, with a follow-up phone call to 30 participants one month later to assess test–retest reliability. This time frame is considered a rational concession between recollection bias and any changes in the patient’s health status since a very short time interval may affect the patient’s responses due to memory or mood. Findings above 0.9 are considered excellent reliability, 0.8 to 0.9 good reliability, 0.7 to 0.8 acceptable reliability, and 0.6 to 0.7 questionable reliability [16].

Participants completed the Hippocratic heart failure self-care scale, and their demographic characteristics, including age, sex, level of education, and occupational and marital status, were collected. The scale was well received, with participants reporting that it was clear, relevant, and easy to complete within 5–10 min.

### 2.4. Ethics

Informed consent was obtained from all subjects involved in the study, and ethical approval was obtained by the Ethical Committee. The study was conducted according to the principles outlined in the Declaration of Helsinki, and anonymity and confidentiality were guaranteed.

### 2.5. Statistics

The mean, standard deviation (SD), median, and interquartile range were used to describe the quantitative data, whereas percentage (%) and frequencies (N) were used for qualitative variables. Reliability coefficients measured by Cronbach’s alpha were calculated for the Hippocratic heart failure self-care scale to assess the reproducibility and consistency of the instrument. A Cronbach coefficient alpha value of >0.59 and <0.95 was considered acceptable [17,18]. The underlying dimensions of the scale were checked with an explanatory factor analysis using a Varimax rotation and the principal component method as a usual descriptive method for analyzing grouped data. A factor analysis, using a principal component analysis with a Varimax rotation, was carried out to determine the dimensional structure of the Hippocratic heart failure self-care scale using the following criteria: (a) eigenvalue > 1; (b) variables should load > 0.50 on only one factor and less than 0.40 on other factors; (c) the interpretation of the factor structure should be meaningful; and (d) the scree plot is accurate if the means of commonalities are above 0.60. A Bartlett’s test of sphericity with *p* < 0.05 and a Kaiser–Meyer–Olkin (KMO) measure of sampling adequacy of 0.6 were used in performing this factor analysis. A factor was considered important if its eigenvalue exceeded 1.0 [19].

A correlation analysis was used to assess internal consistency reliability. The correlation coefficient must not be negative or below 0.20. For qualitative and quantitative steps on the attitude scale’s development, Pearson’s rank correlation coefficient was used to measure the level of agreement between responses at the test and retest. In addition, a linear regression model with the level of adherence as the dependent variable and one independent variable (such as socioeconomic factors and the relationship between patients and healthcare providers) was used to assess the relationship between the level of adherence and the added independent variable. The level of significance was 0.05. The analysis was conducted via SPSS 22.0.

## 3. Results

The demographic characteristics of the participants are shown in Table 2. The demographic characteristics of the sample indicated that 52.8% were women with a mean age of 70 years (SD = 46.43). Most of the participants were divorced or widowed (85.6%), 43.6% had a higher educational level, and 26.0% were employed. Over half of the patients had NYHA III CHF. Common comorbidities included diabetes mellitus (25.2%) and respiratory disease (16.8), with coronary artery disease as the primary cause of CHF (Table 3).

The Hippocratic heart failure self-care scale demonstrated sufficient reliability, with a Cronbach’s alpha of 0.906 for the whole scale (Items 1–22). The subgroup analyses also indicated reliability for men (0.79), women (0.82), NYHA II (0.73), NYHA III (0.85), and NYHA IV (0.80).

The KMO measure of sampling adequacy was 0.658, and Bartlett’s test of sphericity was 1971.02, with df = 142 at *p* < 0.001. The factor analysis identified two primary factors: “medication aspects” and “diet aspects”, which explained 88.44% of the entire variance, as presented in Table 4. The first one encompassed items related to medication: 1 (forget to take medication), 2 (omit to take medication due to its side effects), 3 (omit to take medication when patients feel better), 4 (omit to take medication when patients are outside/travel), and 17 (change the doses according to recommendations); this was termed “medication aspects”. The second factor includes the following items: 5 (daily consumption of fruit and vegetables), 6 (consumption of food responsible for weight increase), 7 (consumption of salty food), 8 (shake salt on your food), 9 (read food labels for ingredients), and 10 (adaption of liquid consumption); this was termed “diet aspects”. Cronbach’s alpha was 0.702 for “medication aspects” and 0.251 for “diet aspects”.

**Figure 1:**
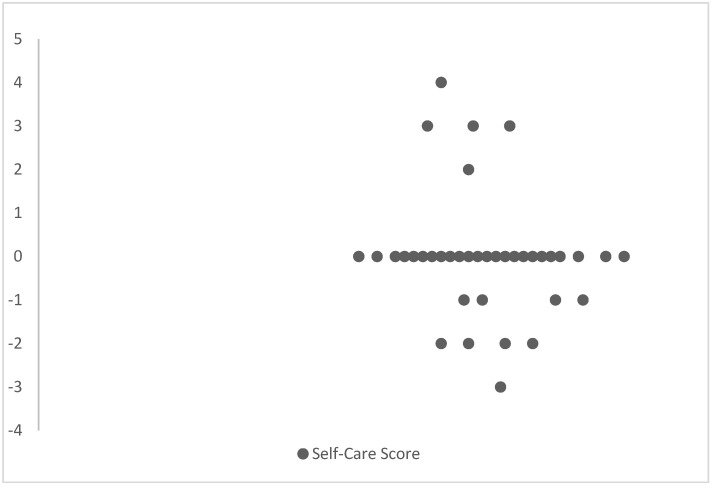
Bland and Altman Method Scatter Plot.

The Hippocratic heart failure self-care scale exhibited strong stability over time, with a high positive correlation (r = 0.973, *p* < 0.001) in the test–retest reliability assessment. A Bland and Altman Method Scatter Plot and the Cohen Kappa statistic further demonstrated strong inter-rater reliability and agreement between measurements (Figure 1).

The Hippocratic heart failure self-care scale was well shouldered by the individuals since it was not difficult and required less than 10 min to be answered. The items were assessed as pertinent, sensible, and plain. On account of that, the face validity was considered very good. The test–retest analysis indicates a high positive correlation between the total scores of the assessments (r = 0.983; *p* < 0.001). The total score on the Hippocratic heart failure self-care scale was significantly lower among patients with NYHA IV (*t* = 2.298; *p* = 0.026). In addition, the scores for the medication and diet sub-scales were significantly higher among participants with NYHA IV (*p* > 0.05). According to the correlation analysis, the level of self-care was not related to age (r = −0.761; *p* > 0.05), gender (*t* = 0.317; *p* > 0.05), or education level (*p* > 0.05). However, the total score on the Hippocratic heart failure self-care scale was associated with the presence of comorbidities. For instance, the level of self-care was lower among patients with diabetes mellitus and respiratory or kidney disease than other patients without comorbidities (*p* < 0.01). The main differences were observed in the sub-scales of medication and symptoms.

## 4. Discussion

This is a clinical study that aimed to assess the development and consequent validation of a self-care assessment tool, namely the Hippocratic heart failure self-care scale. The Hippocratic heart failure self-care scale is a disease-specific tool for assessing the level of self-care in patients with CHF. The Cronbach’s alpha was 0.906 for the entire scale based on the validation analysis, whereas the factor analysis detected two main factors. Further analysis did not show a satisfactory Cronbach’s alpha for these two factors. These domains accounted for 88.44% of the total variance.

According to the latest guidelines of the European Society of Cardiology, self-care recommendations for patients with heart failure concern nutrition, physical activity, medication adherence, psychological status, sleep, leisure and travel, smoking, immunization, symptom monitoring, and management [20]. This study marks the first attempt to develop a comprehensive tool for evaluating self-care behaviors in patients with heart failure, which holds significant potential for integration into both research and clinical practice. For instance, the Self-Care Assessment Schedule (SCAS), Self-Care Behavior Questionnaire, and Self-Care in Chronic Illness Questionnaire are non-disease-specific questionnaires assessing some aspects of self-care among patients with chronic health diseases [2,9,10]. The Beliefs About Medication Compliance Scale and the Beliefs About Dietary Compliance Scale are two disease-specific tools assessing only self-care behavior regarding medicines and diet among patients with CHF [11]. The Self-Care of Heart Failure Index estimates self-care behaviors like medications, diet, and symptom management, whereas the Revised European Heart Failure Self-Care Behavior Scale does not consider the recognition of signs and symptoms of deterioration of heart failure and immunization [13].

The validation study indicated very good internal consistency for the entire scale, although the sub-scales related to “diet”, “alcohol”, “appointment keeping”, and “vaccination aspects” exhibited low Cronbach’s alpha values. The “smoking” and “exercise” sub-scales each had only one question, precluding the calculation of Cronbach’s alpha. The “symptoms” and “medications” sub-scales had Cronbach’s alpha values of 0.506 and 0.702, respectively. Therefore, the scale is suggested to be used as an entire tool.

The factor analysis identified two factors, “medication aspects” and “diet aspects”, which may provide valuable insights into self-behaviors among CHF patients. The scale offers healthcare providers the ability to categorize patient adherence into “very good”, “good”, “fair”, and “poor” levels based on score quartiles, facilitating targeted interventions.

The test–retest reliability results suggest that the Hippocratic heart failure self-care scale is stable over time, indicating its potential for the long-term monitoring and assessment of patient self-behaviors. This is further supported by the strong agreement between the measurements observed in the Bland and Altman Method Scatter Plot and the Cohen Kappa statistic.

The Hippocratic heart failure self-care scale offers a valuable tool for clinical practice, enabling healthcare providers to identify patients who may benefit from interventions aimed at improving their self-behavior. Further studies with a larger population or even controls from two different populations would be useful. Future research should involve cross-sectional and cohort studies to educate clinical practitioners and guide interventions for self-care behaviors in CHF patients. This point is negotiated in the latest guidelines of the European Society of Cardiology, which mention the need for further clinical recommendations to healthcare providers for the best management of heart failure [19].

Our study had some limitations. The Hippocratic heart failure self-care scale is a self-administered tool; therefore, information bias could affect the results. Also, due to the lack of a gold-standard tool, the research team could not conduct an ROC analysis.

## 5. Conclusions

The Hippocratic heart failure self-care scale had satisfactory reliability, and the factor analysis indicated two main factors that were of interest. Therefore, we can state that it is a reliable and valid scale for assessing self-care behaviors in people with heart failure. The score of the scale is independent of the demographic characteristics of patients with heart failure; therefore, it could be used for any patient with heart failure without any limitations. Healthcare providers can use it in their clinical practice to enhance the identification of patients who do not follow and adopt the recommended self-care behaviors. Future studies are recommended to inform clinical practitioners and guide the development of specific interventions for self-care behaviors in patients with CHF.

## Figures and Tables

**Table 1:** The Hippocratic heart failure self-care scale.

**Item**	**How Often during the Last Month:**	**Never**	**Rarely**	**Sometimes**	**Very Often**	**Always**
Q1	Forget to take your heart failure medicine?	0	1	2	3	4
Q2	Did you decide not to take your heart failure medicine because you got side effects from a drug?	0	1	2	3	4
Q3	Did you miss taking your heart failure pills when you felt better?	0	1	2	3	4
Q4	Did you forget to take medicine when you traveled or left home?	0	1	2	3	4
Q5	Did you eat daily fruit and vegetables?	0	1	2	3	4
Q6	Did you eat foods responsible for body weight increase?	0	1	2	3	4
Q7	Did you eat salty foods?	0	1	2	3	4
Q8	Did you shake salt on your food before you ate it?	0	1	2	3	4
Q9	Did you read the labels on foods regarding salt and fat content?	0	1	2	3	4
Q10	Did you change the liquid consumption according to the instructions of your doctor?	0	1	2	3	4
Q11	Did you omit exercise according to the recommended instructions?	0	1	2	3	4
Q12	Did you stop exercising due to dyspnea or palpitation feeling?	0	1	2	3	4
Q13	Did you consume alcohol (scotch, vodka, etc.) daily: more than 2 units for men and 1 unit for women?	0	1	2	3	4
Q14	Did you consume daily more than 2 glasses of wine or 2 beer cans for men and 1 glass of wine or 1 beer can for women?	0	1	2	3	4
Q15	Did you smoke?	0	1	2	3	4
Q16	Did you omit the daily body weight measurement?	0	1	2	3	4
**Item**	**How often during the last year:**	**Never**	**Rarely**	**Sometimes**	**Very Often**	**Always**
Q17	Did you change the dose of diuretics regarding your body weight according to the instructions of your doctor?	0	1	2	3	4
Q18	Did you call your doctor/nurse in case of an increase in your body weight above 2 kg in 3 days?	0	1	2	3	4
Q19	Did you miss a scheduled appointment with your physician/nurse?	0	1	2	3	4
Q20	Did you miss the scheduled appointment for medical examinations?	0	1	2	3	4
**Item**	**In general**	**Never**	**Rarely**	**Sometimes**	**Very Often**	**Always**
Q21	Did you miss the annual vaccination?	0	1	2	3	4
Q22	Did you miss the vaccination for the pneumococcus as suggested?	0	1	2	3	4

**Table 2:** Demographic characteristics of patients.

Characteristic	*n* (%)
Gender	
Male	118 (47.2)
Female	132 (52.8)
Age (years) (SD)	70.5 (46.43)
Education level	
Compulsory	80 (32.0)
Intermediate	61 (24.4)
University	109 (43.6)
Marital status	
Married	23 (9.2)
Divorced/widower	214 (85.6)
Unmarried	13 (5.2)
Living conditions	
Alone	21 (8.4)
Family/relation/other support network	229 (91.6)
Employment status	
Employed	65 (26.0)
Unemployed	121 (48.4)
Retired	43 (17.2)
Household	21 (8.4)

SD: Standard Deviation.

**Table 3:** Clinical characteristics and habits of patients.

Characteristic	*n* (%)
Cause of congestive heart failure	
Coronary artery disease	107 (42.8)
Cardiomyopathy	31 (12.4)
Heart valve disease	19 (7.6)
Congenital heart disease	19 (7.6)
Comorbidity	
Diabetes mellitus	63 (25.2)
Arterial hypertension	8 (3.2)
Respiratory disease	42 (16.8)
Kidney disease	31 (14.8)
Classification of heart failure according to NYHA	
II	71 (28.4)
III	128 (51.2)
IV	51 (20.4)
Left ventricular ejection fraction (%)	29.68 (1.48)
Smoking	
Yes	50 (20.0)
Daily alcohol consumption	
Yes	26 (10.4)

NYHA: New York Heart Association.

**Table 4:** Exploratory factors and explained variance after rotation for the Hippocratic heart failure self-care scale.

Factors											Rotation Sums of Squared Loadings		
		Rescaled Loading	Eigenvalues								% of Variance	Cumulative Variance	Cronbach’s Alpha
			Factor 1	Factor 2	Factor 3	Factor 4	Factor 5	Factor 6	Factor 7	Factor 8			
Factor 1	Question 1	0.801	0.363	0.201	0.602	0.502	0.178	0.201	0.198	0.193	68.02	68.02	0.702
	Question 2	0.887	0.103	0.565	0.198	0.306	0.630	0.206	0.025	0.497			
	Question 3	0.896	0.598	0.301	0.524	0.486	0.211	0.096	0.168	0.276			
	Question 4	0.798	0.804	0.185	0.054	0.369	0.199	0.143	0.062	0.303			
Factor 2	Question 5	0.802	0.678	0.152	0.295	0.481	0.020	0.031	0.143	0.159	20.42	88.44	0.251
	Question 6	0.693	0.332	0.270	0.589	0.078	0.263	0.100	0.219	0.283			
	Question 7	0.753	0.515	0.355	0.261	0.328	0.232	0.123	0.415	0.053			
	Question 8	0.722	0.445	0.088	0.102	0.378	0.348	0.406	0.360	0.303			
	Question 9	0.820	0.410	0.720	0.040	0.130	0.265	0.214	0.079	0.066			
	Question 10	0.225	0.370	0.017	0.065	0.067	0.057	0.774	0.342	0.248			
Factor 3	Question 11	0.817	0.316	0.021	0.452	0.072	0.330	0.621	0.232	0.187	2.55	90.51	
Factor 4	Question 13	0.822	0.498	0.039	0.030	0.745	0.027	0.210	0.126	0.101	3.50	96.159	0.208
	Question 14	0.732	0.336	0.150	0.032	0.111	0.536	0.261	0.464	0.161			
Factor 5	Question 15	0.858	0.309	0.382	0.290	0.180	0.654	0.245	0.126	0.079	1.05	97.20	
Factor 6	Question 12	0.698	0.495	0.082	0.131	0.435	0.146	0.287	0.169	0.468	3.43	99.20	0.560
	Question 16	0.875	0.901	0.010	0.082	0.016	0.055	0.036	0.067	0.056			
	Question 17	0.556	0.938	0.027	0.109	0.018	0.141	0.005	0.149	0.054			
	Question 18	0.933	0.021	0.840	0.321	0.118	0.254	0.159	0.195	0.002			
Factor 7	Question 19	0.846	0.186	0.609	0.496	0.200	0.135	0.243	0.302	0.001	0.62	99.83	0.057
	Question 20	0.639	0.810	0.039	0.058	0.069	0.203	0.127	0.027	0.245			
Factor 8	Question 21	0.936	0.021	0.840	0.321	0.118	0.254	0.159	0.195	0.002	0.41	100.00	0.430
	Question 22	0.873	0.059	0.427	0.663	0.214	0.400	0.140	0.203	0.059			

## Data Availability

Data are contained within the article.
